# *ContaMiner* and ContaBase: a webserver and database for early identification of unwantedly crystallized protein contaminants

**DOI:** 10.1107/S1600576716014965

**Published:** 2016-11-02

**Authors:** Arnaud Hungler, Afaque Momin, Kay Diederichs, Stefan, T. Arold

**Affiliations:** a King Abdullah University of Science and Technology (KAUST), Center for Computational Bioscience Research (CBRC), Division of Biological and Environmental Sciences and Engineering (BESE), Thuwal, 23955-6900, Saudi Arabia; b Fachbereich Biologie, Universität Konstanz, M647, D-78457 Konstanz, Germany

**Keywords:** diffraction, molecular replacement, contaminant crystals, computer programs

## Abstract

Solving the phase problem in protein X-ray crystallography relies heavily on the identity of the crystallized protein, especially when molecular replacement (MR) methods are used. Yet, it is not uncommon that a contaminant crystallizes instead of the protein of interest. Such contaminants may be proteins from the expression host organism, protein fusion tags or proteins added during the purification steps. Many contaminants co-purify easily, crystallize and give good diffraction data. Identification of contaminant crystals may take time, since the presence of the contaminant is unexpected and its identity unknown. A webserver (*ContaMiner*) and a contaminant database (ContaBase) have been established, to allow fast MR-based screening of crystallographic data against currently 62 known contaminants. The web-based *ContaMiner* (available at http://strube.cbrc.kaust.edu.sa/contaminer/) currently produces results in 5 min to 4 h. The program is also available in a github repository and can be installed locally. *ContaMiner* enables screening of novel crystals at synchrotron beamlines, and it would be valuable as a routine safety check for ‘crystallization and preliminary X-ray analysis’ publications. Thus, in addition to potentially saving X-ray crystallographers much time and effort, *ContaMiner* might considerably lower the risk of publishing erroneous data.

## Abstract 2

A webserver, titled *ContaMiner*, has been established, which allows fast molecular-replacement-based screening of crystallographic data against a database (ContaBase) of currently 62 potential contaminants. *ContaMiner* enables systematic screening of novel crystals at synchrotron beamlines, and it would be valuable as a routine safety check for ‘crystallization and preliminary X-ray analysis’ publications.

## 1. Introduction   

Apart from some *in vivo* produced crystals (Koopmann *et al.*, 2012 ▸; Sawaya *et al.*, 2014 ▸; Gallat *et al.*, 2014 ▸), protein X-ray crystallography requires purification of the protein of interest. Although purification steps include selection according to an affinity for specific functional groups (*e.g*. nickel or gluta­thione), followed by selection for surface charges and/or molecular weight, it is not uncommon that protein contaminants co-purify (Bolanos-Garcia & Davies, 2006 ▸; Psakis *et al.*, 2009 ▸; Niedzialkowska *et al.*, 2016 ▸). Even membrane protein purification methods are prone to contamination (Veesler *et al.*, 2008 ▸; Psakis *et al.*, 2009 ▸; Kors *et al.*, 2009 ▸). Indeed, several bacterial proteins were shown to bind to nickel beads, even without a polyhistidine tag (Bolanos-Garcia & Davies, 2006 ▸; Tiwari *et al.*, 2010 ▸; Kozlov *et al.*, 2013 ▸). Other possible contaminants include proteins added to facilitate protein purification (*e.g.* lysozyme, DNase or proteases) or proteases added to facilitate crystallization through *in situ* proteolytic cleavage (Dong *et al.*, 2007 ▸; Wernimont & Edwards, 2009 ▸). Moreover, protein fusion tags (Costa *et al.*, 2014 ▸) might either be expressed without the protein of interest or co-purify after their proteolytic cleavage (Niedzialkowska *et al.*, 2016 ▸). Even antibiotic resistant bacteria that persist within *Escherichia coli* cultures can be an unexpected source of contamination (Keegan *et al.*, 2016 ▸). Currently known contaminants have a wide range of molecular weights, from 10 to 140 kDa (see the supporting information). Most contaminants weigh between 20 and 40 kDa, which also corresponds to the weight range of ∼70% of all crystallized proteins reported in the Protein Data Bank (PDB). Contaminants with a similar molecular weight to most proteins of interest can therefore co-elute during size exclusion chromatography and are not identifiable on SDS PAGE gels. Moreover, contaminants that appear as minor bands on gels may still be forming crystals instead of the targeted protein (*e.g.* Benini *et al.*, 2004 ▸; Hu *et al.*, 2007 ▸; Kozlov *et al.*, 2013 ▸; Niedzialkowska *et al.*, 2016 ▸). Once crystals have been obtained, only a mass spectrometric analysis on thoroughly washed crystals might be able to reveal that a contaminant has crystallized. However, this test is frequently omitted for reasons of feasibility in terms of size, number or stability of crystals, and access to the required instrument.

If the wrong identity is assumed for the crystallized protein, the solution of the crystallographic phase problem is highly likely to fail because X-ray structure determination relies heavily on the identity of the protein. Especially in molecular replacement (MR), the most frequently used method, the protein sequence is needed to identify structural templates and to evaluate the number of molecules expected in the asymmetric unit (ASU) (Evans & McCoy, 2008 ▸; Rossmann & Blow, 1962 ▸). Isomorphous replacement methods also use the protein sequence, for example to assess the number of residues capable of fixing or incorporating particular heavy atoms (*e.g.* cysteines, methionines), or the number of non-crystallographic symmetry-related molecules in the ASU. Since the lack of success in solving the phase problem can have many reasons (including indexing errors, twinning, anisotropy, poor MR template or poorly fixed/highly mobile heavy atoms), crystal formation by a contaminant is not necessarily suspected. Contaminant crystals can therefore entail loss of much effort and personal or synchrotron time. Many cases might finally be resolved [*e.g.* PDB accessions 4bfl (M. Gabrielsen, A. W. Schuttelkopf & S. N. Akpunarlieva, to be published) or 5jk4 (Keegan *et al.*, 2016 ▸)]. However in the absence of a solution, contaminant crystals might be published erroneously in ‘crystallization and preliminary X-ray analysis’ publications, which may then mislead other researchers in the field unless corrected (Benini *et al.*, 2004 ▸; Hu *et al.*, 2006 ▸). In extreme cases, diffraction data from a contaminant can be used to produce an erroneous model structure of the protein of interest, and be published as a novelty despite very poor refinement statistics (Weiss *et al.*, 2016 ▸).

Even if a contaminant crystal is suspected, there is currently no method to rapidly screen diffraction data against a database of plausible contaminants. The only available tests are based on comparing the space group and cell parameters of a newly obtained crystal with those of published contaminants (Niedzialkowska *et al.*, 2016 ▸). However such simple tests are insufficient, because contaminant proteins that crystallize easily can crystallize in different space groups. For example, for the *E. coli* inorganic pyrophosphatase PPA (P0A7A9) 19 structures are available in the PDB; PPA has been found to crystallize in four different space-group/unit-cell combinations, and even within the same crystal lattice and space group up to 4 Å differences in a single cell parameter were reported. For proteins such as thrombin or lysozyme, for which several hundreds of structures have been reported, many space groups and lattices appear possible. Moreover, the PDB might not contain the complete arsenal of possible crystal forms because there is little incentive for a researcher to refine and deposit yet another contaminant structure, especially if diffraction data are of low resolution. Conversely, with the number of known contaminants expanding rapidly, the likelihood that a true protein of interest crystallizes in a space group with cell dimensions similar to a contaminant becomes substantial. The only possible alternatives are to try MR for each of the possible contaminants, or to run computationally expensive brute-force methods on supercomputers (Stokes-Rees & Sliz, 2010 ▸; Keegan *et al.*, 2016 ▸). We have therefore programmed a publicly available webserver, named *ContaMiner*, which uses parallel MR to screen diffraction data against a hand-curated database (ContaBase) of known contaminants.

## 2. Strategy   

*ContaMiner* consists of a set of scripts and uses the ContaBase database to perform and analyse large-scale MR on experimental X-ray crystallography diffraction data. The scripts are programmed for parallel processing on a cluster or supercomputer to maximize the processing speed and efficiency. Given experimental diffraction data *ContaMiner* simultaneously runs MR trials using all selected proteins from ContaBase (described below).

For MR, *ContaMiner* uses the *MoRDa* automatic molecular replacement pipeline (Vagin & Lebedev, 2015 ▸). Automatic structure solution by *MoRDa* comprises the preparation of templates (based on the *MoRDa* model database), then MR trials using the program *Molrep* (Vagin & Teplyakov, 1997 ▸), then refinement of solutions using *Refmac* (Vagin & Teplyakov, 1997 ▸), and finally assigning a score to each solution. We chose *MoRDa* because of the following features. *MoRDa* requires only minimal input, is highly parallelizable, features a sophisticated algorithm for search model preparation from a regularly updated database and, importantly, can be used in a two-step process: *morda_prep* prepares the MR search models and program parameters, and *morda_solve* runs the MR and refinement step based on these models and parameters. These features allow *ContaMiner* to prepare and optimize MR search models and program parameters for all contaminants prior to data submission by users. MR searches then involve only *morda_solve*, enhancing the speed of processing. Further optimization and machine-learning capacities were implemented to accelerate the MR process, as described below.

The *ContaMiner* output summarizes the *MoRDa* scores from all MR trials, and infers the likelihood of each individual contaminant being a solution. For each likely solution, the user can download the *morda_solve* log file, the MR-positioned model in PDB format and the MTZ-format data file with calculated phases, based on the refined MR model.

## 3. ContaBase, the contaminant database   

We have established and curated ContaBase, a database of currently 62 contaminants. ContaBase was compiled from publications, postings on the CCP4 Bulletin Board, (Murray, 2010 ▸; Wojdyr, 2016 ▸), errata of ‘preliminary X-ray diffraction’ reports (Benini *et al.*, 2004 ▸; Hu *et al.*, 2006 ▸) and orally communicated contaminants (see the full listing in the supporting information). These contaminants stem from the protein expression host organism (currently 35 from *E. coli*, two from yeast) and from an antibiotic resistant bacterial contaminant of *E. coli* expression cultures (*Stenotrophomonas maltophilia*) (Keegan *et al.*, 2016 ▸). Contaminants also include commonly used fusion tags for affinity purification and proteins that are frequently added as part of the purification protocol (Stokes-Rees & Sliz, 2010 ▸; Bolanos-Garcia & Davies, 2006 ▸; Psakis *et al.*, 2009 ▸; Veesler *et al.*, 2008 ▸; Kors *et al.*, 2009 ▸; Costa *et al.*, 2014 ▸; Tiwari *et al.*, 2010 ▸; Kozlov *et al.*, 2013 ▸; Niedzialkowska *et al.*, 2016 ▸; Wernimont & Edwards, 2009 ▸; Kiser *et al.*, 2007 ▸).

ContaBase can be downloaded as an XML file, and is included in the set of scripts available on the github repository (see §4). Through a ‘contact us’ tab on the *ContaMiner* web page, users are requested to suggest additional relevant proteins that are missing from the current database. For 53 out of 62 contaminants there are 80–100% identical three-dimensional structures available (supporting information). For seven out of the remaining nine contaminants, the available crystallographic MR templates have 33–66% sequence identity, which suggests that MR is still highly likely to succeed. For two plausible contaminants (α-galactosidase and acetyl­ornithine de­acetyl­ase) only ∼25% identical templates exist, which indicates that these MR cases might not always be successful.

## 4. Implementation and programming   

*ContaMiner* scripts are written in the Shell command language (sh) and are POSIX compliant. The scripts have the following dependencies: *SLURM* (Yoo *et al.*, 2003 ▸), *CCP4* (Collaborative Computational Project, Number 4, 1994 ▸) and *MoRDa* (Vagin & Lebedev, 2015 ▸). The scripts use the common utilities *procmail*, *sed*, *wget* and *xmllint. ContaMiner* is a free software distributed under GNU General Public License v2. A github repository is available at https://github.com/StruBE-KAUST/ContaMiner. The *ContaMiner* web interface (§7) is designed in Python, and uses Django as a web framework. The source code is also available on github (https://github.com/StruBE-KAUST/django-contaminer). Local installations of *Conta­Miner* require at least 400 MB of space disk, plus 5.8 GB for *CCP4* and *MoRDa*. While *ContaMiner* can be implemented on a 600 MHz single-core Pentium III, it is recommended to have as many floating-point units (FPUs) as required to run each MR job in parallel (∼2150, or less if not all contaminants are selected).

In *ContaMiner*, MR templates are prepared by *morda_prep* once, prior to their subsequent parallel use for all submitted MTZ files. *Morda_prep* chooses one or more PDB files from its database to prepare the MR search models according to sequence identity, resolution and completeness of the PDB model. To enhance the success of the MR procedure, *morda_prep* may prepare several search models from each PDB template, typically comprising the protein monomer (truncated according to sequence differences and similarities), but also (sub-)domains, ensemble models and/or plausible multimers. Thus, flexibility between domains/lobes or high copy numbers in the ASU should not prevent the success of MR. One or more search models form a model ‘pack’, which is then used as a simultaneous search unit by *morda_solve* (Vagin & Lebedev, 2015 ▸). *Morda_prep* produces automatically different search models for each sequence in ContaBase. For this, *morda_prep* uses its own curated database of representative structures, which contains a sufficient number of representative structures to cover the structures available in the PDB for use in MR. It is therefore possible that *morda_prep* uses a ∼90% identical homologue as template for a contaminant sequence for which a 100% identical PDB structure exists (this is the case, for example, for CAN_ECOLI and IPYR_ECOLI; see supporting information). Given the low success rate of NMR models in MR, *morda_prep* uses homologous crystal structures even if 100% identical NMR models exist (*e.g.* the case of SLYD_ECOLI, where ∼50% identical homologues are used). Our tests, and tests submitted by users (see below), indicate that this behaviour of *MoRDa* does not preclude the detection of these contaminants by *ContaMiner*.

In *ContaMiner*, running one job consists of launching one instance of *morda_solve* per alternative space group (from the input data file), per pack. Each single instance of *morda_solve* produces a log file that also contains the percentage and the Q_factor scores (Keegan *et al.*, 2011 ▸), which evaluate the quality of the final model. By collecting the percentage and the Q_factor scores for every single instance of *morda_solve*, *ContaMiner* produces a result file, named results.txt, which summarizes the processes. Each single line corresponds to an instance of *morda_solve*. A line follows this format:

XXXXXX\_Y\_Z-Z-Z-Z:state:time

where XXXXXX is the UniProt ID (The UniProt Consortium, 2015 ▸) of the contaminant, Y is the number of the pack (according to the output of *morda_prep* or the table in the supporting information), Z-Z-Z-Z is the space group, state is the state of the process and time is the time spent to run this specific process. Here, state can be one of the following:

(*a*) cancelled: if the process did not run at all

(*b*) aborted: if the process has been stopped before the end

(*c*) error: if *morda_solve* encountered an error

(*d*) nosolution: if the *morda_solve* solution has a Q_factor below Q_lim (0.4) (Vagin & Lebedev, 2015 ▸)

(*e*) A-B: if *morda_solve* found a solution with Q_factor > Q_lim, where

(i) A is the Q_factor given by *morda_solve* for this solution (Keegan *et al.*, 2011 ▸)

(ii) B is the percent score given by *morda_solve* for this solution

time can be 0h 0m 0s, meaning the job was cancelled without being launched, or a non-null value displaying how long the process was running before completion or abortion.

**Figure 1:**
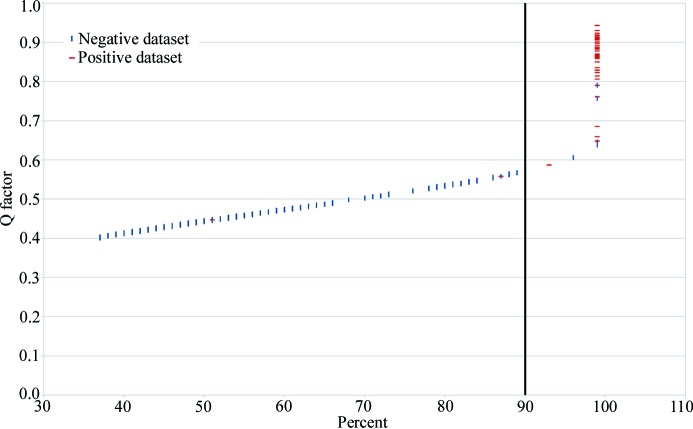
Evaluation of the *ContaMiner* cutoff criteria. *MoRDa* Q_factor and percent scores were plotted for 45 positive (matching) and 530 negative (not matching) PDB/data file pairs. The percent score of 90% was empirically chosen as a cutoff value for positive results (*i.e.* contaminant present) on the basis of this analysis. The outlier positive control with a percent score of 52% is PDB ID 1f6m (Lennon *et al.*, 2000 ▸), a 3 Å-resolution complex of ∼1700 residues, with four molecules of thio­redoxin (108 residues each).

To determine which score and value are best to indicate that a contaminant is present, we launched *MoRDa* 575 times with different models and sequences from two different datasets. The first dataset contained 45 pairs of matching models and sequences (positive dataset), while the second contained 530 non-matching pairs of models and sequences (negative dataset). For each best MR solution found, the percent and Q_factor scores given by *MoRDa* were plotted as *x* and *y* axis coordinates, respectively, in Fig. 1 ▸. The separation between positive and negative dataset was more pronounced for the percent scores than for the Q_factor. We therefore chose the percent score as evaluation criterion, rather than the Q_factor. As the purpose of *ContaMiner* is to alert the user about the possible presence of a contaminant, we chose a percent cutoff limit for a positive result at 90%, which resulted in a low false negative rate of 4% (two wrong results over 45 tests) while keeping the false positive rate at 0.8% (three wrong results over 530 tests). Lowering the limit below 90% decreases the false negative rate only marginally, while substantially increasing the false positive rate. Conversely, increasing the limit would entail many more false negatives.

## 5. Optimization   

Depending on the number of contaminants (which can be reduced by deselecting irrelevant ones), the number of packs per contaminant and the number of alternative space groups to test, *ContaMiner* may run approximately 2000 processes. The maximum number of processes occurs when all contaminants are tested, and when the provided experimental data are in a space group equivalent to *P*222. Such a job requires at the moment ∼260 packs to be tested in eight different space groups, which means *ContaMiner* runs just under 2100 processes. This number will increase as more contaminants are included in ContaBase. MR takes significantly longer for a particular process if no solution is found. On our current cluster with 2.30 GHz CPU, *morda_solve* may return some positive results after only 5 min, but it may require up to 48 h per process which has no solution. The following features were implemented to optimize and accelerate the process.

*Stop other processes if a contaminant is detected*. Since it is highly unlikely that two different contaminants will be found in the same crystal, it is unnecessary to continue processing all contaminants of the database if one contaminant is detected with a *MoRDa* percent score of 90% or above. Therefore, in such a case, all processes are stopped. This optimization is ineffective if the result is negative throughout (no contaminant found). However, if the job has a positive MR result, *ContaMiner* stops the remaining processes rather than waiting for the negative trials to finish. Since in most of the positive cases a solution is found in less than 35 min, this procedure frees many CPUs for the following submissions and reduces the response time considerably. As a partial exception to this rule, *ContaMiner* does not stop processes of contaminants that are sufficiently similar in three-dimensional structure to allow solving one contaminant with the three-dimensional structure of another. Currently this is only the case for the family of structural homologues ENTK_BOVIN, FA10_HUMAN, TRY1_BOVIN, CTRA_BOVIN and DNAS1_BOVIN (all of which are agents added during protein preparation). In this particular case, detection of a positive result for one of these will not stop processing of the other structural homologues (but all remaining processes of the detected contaminant will still be stopped).

*Stop all processes after 4 h*. In none of the tests (see below) did *ContaMiner* return positive results after 3 h. We have therefore set the maximum duration time to 4 h. After 4 h all remaining jobs will be killed or cancelled, and a negative result communicated. With more data available, we expect to shorten this limit further in future versions.

*Machine learning; most likely comes first*. Not all contaminants and not all alternative space groups are equally likely. For example *P*2_1_2_1_2_1_ occurs much more frequently (22% of all PDB entries) than *P*222 (0.01%) (see http://www.rcsb.org/pdb/static.do?p=general_information/pdb_statistics/). Given that *ContaMiner* abrogates the search if solutions are detected, prioritizing helps in shortening the overall calculation time. A simple form of machine learning enables *ContaMiner* to learn the likelihood of a particular model of a contaminant and space group as being a solution through its successes in previous runs. When a new job is submitted, the combination of space group and model are sorted thanks to a likelihood matrix, then submitted in this order. Currently, initialization is done on the basis of the space group likelihood distribution from the PDB depositions. All packs of all contaminants have initially an equal likelihood. For each successful MR, the likelihood for the successful contaminant is incremented. The *ContaMiner* job submission matrix is automatically updated with each positive result, thus ultimately prioritizing most likely contaminants. In the case of equal likelihood, the job selection follows the lexicographic order, first by UniProt ID of the contaminant, then number of the model, then space group.

## 6. Performance testing   

*ContaMiner* is tested before each release. For the current release we conducted a total of 100 blind tests. As positive controls, we used the diffraction data for 53 contaminants that are currently available in the PDB. For testing a specific contaminant, the diffraction data files were randomly taken from the ‘Structure’ section of the UniProt database, without checking if the associated structural model was used as template for the MR search models prepared by *morda_prep*. The remaining 47 (out of 100 tests) consisted of negative controls. These structure factor files were randomly selected from the PDB, and checked manually to ensure that their structure was not similar to a known structure of a contaminant.

Of the 53 positive controls, 51 gave a *ContaMiner* solution. The remaining two cases (false negatives) are unrealistic examples, where the contaminant is part of a larger complex. In PDB ID 2acz (Horsefield *et al.*, 2006 ▸), the contaminant SdhC (succinate de­hydrogenase cytochrome b556 subunit) contributes only 129 residues to a 1070-residue complex. In 1aon (Xu *et al.*, 1997 ▸), the contaminant GroEL is found in an asymmetric complex with ADP-bound GroES. In this particular assembly the one heptameric GroEL unit in contact with GroES adopts a clearly distinct conformation that is not prepared as a search model by *morda_prep*; only one GroEL heptamer is placed, resulting in percent scores below the 90% cutoff. All the of 47 negative tests gave negative results. The 51 positive cases gave a result in an average time of 35 min. About a quarter of cases finished within 5–10 min, and a single outlier took 2 h 58 min (PDB ID 1scz; a densely packed trimer of ODO2_ECOLI with secondary-structure exchange between protomers; N. Schormann *et al.*, to be published). The negative cases, as well as the false negative cases, all took the maximum allowed 4 h.

Prior to publication, *ContaMiner* had been tested independently by 37 external users who submitted 83 jobs through the web interface (see below). Eight of the submitted jobs gave a positive result [CAN_ECOLI (3×), IPYR_ECOLI, HFQ_ECOLI, RS15_ECOLI, SLYD_ECOLI and B4SL31_STM5]. Of these, CAN_ECOLI and IPYR_ECOLI present cases where *morda_prep* uses ∼90% identical homologues as templates, instead of the available 100% identical PDB structures. SLYD_ECOLI was solved and detected using only ∼50% identical structures as templates. *ContaMiner* also found the correct contaminant in the case of the wrongly reported SMN protein (Weiss *et al.*, 2016 ▸).

## 7. Web interface   

A web interface is available at https://strube.cbrc.kaust.edu.sa/contaminer. The required input is the (merged and scaled) diffraction data file, which can be presented in the MTZ or crystallographic information file (CIF) formats. Other inputs are the user’s email address and optionally a name for the submitted job. To increase speed and preserve resources for other jobs, the user can deselect unlikely contaminants in the ‘Contaminants’ tab. Protein purification tags and proteins that might be added during the purification or crystallization process are deselected by default, and need to be selected by the user if they were used.

A file submitted through this web interface runs *Conta­Miner* on a computer cluster with 19 456 CPUs at 2.30 GHz, with 1 FPU per CPU, at the King Abdullah University of Science and Technology. The jobs are executed on a first-come first-served basis. When the job is completed, users receive an email with a link to the results. For all contaminants tested a positive result (percent score > 90%) is indicated with a green checkmark. Cases where the percent score is below 90%, but the Q_factor is above Q_lim (corresponding to a percent score of about 37%; Fig. 1 ▸), appear with a yellow exclamation mark; these cases are unlikely to be one of the tested contaminants, based on our evaluations. Finally, a red cross indicates that *morda_solve* did not return any solution for this case (nosolution category, with Q_factor < Q_lim). By clicking on a contaminant, the user can obtain a UniProt link and the space group, as well as percent and Q_factor scores of the best MR solution. For positive results (percent score > 90%), PDB, MTZ and log files given by *morda_solve* can be downloaded from within the ‘File’ section of the popover.

If a user-submitted dataset indicates an MR solution for a contaminant for which no identical model is available in the PDB (*i.e.* sequence less than 80% identical or coverage less than 60%), then this is indicated to the user to encourage structure deposition and/or publication. In all cases where positive results are obtained, the user is encouraged to check if the submitted dataset is of a significantly higher resolution than the currently available MR template, which then might justify deposition of the user’s model and data at the PDB.

User-provided MTZ files are deleted as soon as the job is completed and are not made public. The *ContaMiner* statistics, and in the few positive cases the *morda_solve* MTZ and PDB files, remain currently available as long as storage space is available.

## 8. Discussion   

We have established software, a webserver and a database, for the crystallographic community to alleviate dealing with unwantedly crystallized contaminants. *ContaMiner* provides a convenient and rapid test for the presence of contaminant crystals based on experimental diffraction data. The systematic use of *ContaMiner* on unresolved diffraction data may save much time and effort, and should substantially lower the risk of reporting contaminant crystals in ‘crystallization and preliminary X-ray analysis’ publications.

As *ContaMiner* is free open-source software available on github, users can contribute and improve the program, and install it locally for specific uses. ContaBase, the database of known protein contaminants, provides a comprehensive resource for the community, which we hope will be further enriched through continued community input.

Our choice of using the full MR search power on pre-selected contaminants, as opposed to using a full brute-force search optimized for speed, results in certain advantages and disadvantages. The disadvantages are that (i) only contaminants that are known and present in ContaBase are detected; and that (ii) the speed of detection ranges from 5 min to several hours in difficult cases. The advantages are that (i) by providing a carefully curated MR database we minimize the rate of false positives and hence the time spent for manual inspection of all MR solutions produced from non-contaminant structural homologues (especially when data are anisotropic, twinned or of low resolution); and that (ii) the precise MR search with several search models allows detection of contaminants in difficult cases (*e.g.* high copy number in the ASU, twinning, anisotropy, low resolution, protein conformational changes or partial proteolytic cleavage) with a higher success rate than speed-optimized brute-force approaches, or with a higher speed than full MR brute-force approaches. *ContaMiner* and high-speed brute-force algorithms, once they become available, will therefore be highly complementary approaches.

The success of *ContaMiner* is of course limited by all of the factors that hamper MR, including strong anisotropy, twinning, very low resolution and lack of homologous search templates. For two out of the 62 contaminants (α-galactosidase and acetyl­ornithine de­acetyl­ase), the MR template structures currently available in the PDB are less than 30% identical, and solutions may not be identifiable, especially in diffraction data with low resolution or high molecule copy number in the ASU.

Future developments comprise the inclusion of more contaminants from other expression systems (such as insect cells, yeast or other eukaryotic species) into ContaBase. While the current response time of 5 min–4 h already allows users to obtain feedback within an 8 h synchrotron shift (given that new crystal forms are tested early during the beamtime), we intend to implement several additional features to improve the speed. Planned features include a live update of the results while the job is still in progress (a quarter of all positive results appear within 5–10 min), an improved prioritization of the processes based on known contaminant crystal lattices and space groups, and an automated selection of contaminants based on the molecular weight (as optional user-supplied information in the submission form).

## Supplementary Material

Supplementary table in Excel format. DOI: 10.1107/S1600576716014965/ei5009sup1.xls

Supplementary table in pdf format. DOI: 10.1107/S1600576716014965/ei5009sup2.pdf

## Appendix A Contaminants currently included in ContaBase

A list of the contaminants currently included in ContaBase is provided as supporting information. In the table, ‘Identity’ refers to the identity of the templates used by *MoRDa*. As described in §4, *MoRDa* may use templates that are not of the highest possible identity to the contaminant sequence. The references in the table are (1) Niedzialkowska *et al.* (2016 ▸); (2) Bolanos-Garcia & Davies (2006 ▸); (3) Psakis *et al.* (2009 ▸); (4) Veesler *et al.* (2008 ▸); (5) Tiwari *et al.* (2010 ▸); (6) Murray (2010 ▸); (7) personal communication (J. Cooper); (8): personal communication (S. McMahon); (9) personal communication (B. Van Den Berg); (10) own data (KD); (11) Keegan *et al.* (2016 ▸); (12) Costa *et al.* (2014 ▸); (13) Hu *et al.* (2006 ▸).
