# Supplementary material for: ContaMiner and ContaBase: a webserver and database for early identification of unwantedly crystallized protein contaminants
Source: J Appl Crystallogr. 2016 Nov 2;49(Pt 6):2252–8. doi: 10.1107/S1600576716014965 (PMC5140001; doi:10.1107/S1600576716014965)
Supplement: Supplementary file 2 [file j-49-02252-sup2.pdf]

N°	Uniprot ID	Short Name	Full name	Length (AA)	Molecular weight (kDa)	Organism	Pack							Reference
							N°	Template	Chain	Domain	Quaternary structure	Identity (%)	Coverage (%)	
**Proteins from E. coli**														
1	P0ACJ8	CRP_ECOLI	cAMP-activated global transcriptional regulator CRP	210	23.64	*E. coli*	1	3RYP	A		monomer	100	96%	1, 2
							2	3RYP	A	1	domains	100	87%	
								3RYP	A	2		100		
							3	3RYP	AB		dimer	100	96%	
							4	1O3T	B	1	domains	100	86%	
								3KCC	B	2		100		
2	P61517	CAN_ECOLI	Carbonic anhydrase	220	25.10	*E. coli*	1	3QY1	A		monomer	91	98%	1, 2
							2	3QY1	A	1	domain	89	72%	
							3	3QY1	ABCD		tetramer	91	98%	
							4	3QY1	AB		dimer	91	98%	
							5	3QY1	A	1	domains	89	91%	
								3QG5	D	2		29		
3	P0A7A9	IPYR_ECOLI	Inorganic pyrophosphatase	176	19.70	*E. coli*	1	2AU7	A		monomer	90	99%	7
							2	2AU7	A	1	domain	90	82%	
							3	2AU7	ABCD		hexamer	90	100%	
							4	2AU7	ABC		trimer	90	99%	
							5	2AU7	AB		dimer	90	99%	
4	P27302	TKT1_ECOLI	Transketolase 1	663	72.21	*E. coli*	1	2R8O	A		monomer	100	100%	5
							2	2R8O	A	1	domain	100	85%	
								2R8O	A	2	domain	100		
								2R8O	A	3	domain	100		
							3	2R8O	AB		dimer	100	100%	
							4	2R8O	A	1	domain	100	100%	
								2R8O	A	2	domain	100		
								2R8O	A	3	domain	100		
								1W5D	A	2	domain	21		
5	P0A715	KDSA_ECOLI	2-dehydro-3-deoxyphosphooctonate aldolase	284	30.83	*E. coli*	1	1O60	A		monomer	81	95%	5
							2	1O60	A	1	domain	81	81%	
							3	1O60	ABCD		tetramer	81	95%	
							4	1O60	AB		dimer	81	95%	
							5	1O60	AB		dimer	81	95%	
							6	3E9A	A	1	domain	81	93%	
6	P0A9K9	SLYD_ECOLI	FKBP-type peptidyl-prolyl cis-trans isomerase SlyD	196	20.85	*E. coli*	1	4ODK	A		monomer	50	78%	2
							2	4ODK	A	1	domains	52	61%	
								4ODK	A	2		39		
							3	4ODK	AB		dimer	50	78%	
							4	4ODM	B		monomer	50	76%	
							5	4ODM	B	1	domains	54	66%	
								4ODM	B	2		40		
							6	4ODM	AB		dimer	50	76%	
							7	3CGN	A		monomer	49	77%	
							8	3CGN	A	1	domains	54	66%	
								3CGN	A	2		40		
							9	3CGN	A	1	domains	54	84%	
								3LUO	A	1		43		
								2UVO	A	2		26		
7	P69054	DHSC_ECOLI	Succinate dehydrogenase	129	14.30	*E. coli*	1	2WDQ	C		monomer	100	94%	3
							2	2WDQ	C	1	domain	100	84%	
							1	4DX5	C		monomer	100	98%	
							2	4DX5	C	1	domains	100	93%	
								4DX5	C	2		100		
								4DX5	C	3		100		
								4DX5	C	4		100		

								4DX5	C	5		100		
								4DX5	C	6		100		
							3	4DX5	ABC		trimer	100	98%	
8	P31224	ACRB_ECOLI	AcrB; multidrug efflux transporter	1049	113.57	*E. coli*	4	4DX5	AB		dimer	100	98%	3, 4
								2HRT	F	1		100		
								2DRD	A	4		100		
								2I6W	A	3		100		
							5	2GIF	C	6	domains	100	99%	
								2DRD	B	5		100		
								2GIF	A	2		100		
								3G5Q	A	2		100		
							1	1E0T	A		monomer	100	95%	
								1E0T	A	1		100		
							2	1E0T	A	2	domains	99	92%	
								1E0T	A	3		100		
9	P0AD61	KPYK1_ECOLI	Pyruvate kinase I pykF	470	50.73	*E. coli*	3	1E0T	ABCD		tetramer	100	95%	5
							4	1E0T	AB		dimer	100	95%	
								1E0T	A	2		99		
							5	1E0T	A	1	domains	100	97%	
								1E0T	A	3		100		
								5ESV	F	2		29		
							1	1E1O	A		monomer	100	96%	
							2	1E1O	A	1	domains	100	84%	
								1E1O	A	2		100		
10	P0A8N5	SYK2_ECOLI	Lysine--tRNA ligase lysU	505	57.83	*E. coli*	3	1E1O	AB		dimer	100	96%	5
								1E1O	A	2		100		
							4	1E1O	A	1	domains	100	94%	
								1JJ2	O	2		33		
							1	3HY0	A		monomer	99	50%	
							2	3HY0	A	1	domains	98	47%	
								3HY0	A	2		100		
								3HY0	A	1		98		
11	P00957	SYA_ECOLI	Alanine--tRNA ligase alaS	876	96.03	*E. coli*		3HY0	A	2		100		5
								3G98	A			32		
							3	3WQY	A	3	domains	36	96%	
								3FPC	A	1		19		
								2P8W	S			23		
							1	1Z7E	B		monomer	100	97%	
								1Z7E	B	1		100		
							2	1Z7E	B	2	domains	100	93%	
								1Z7E	B	3		100		
12	P77398	ARNA_ECOLI	Bifunctional polymyxin resistance protein ArnA	660	74.29	*E. coli*	3	1Z7E	AB		dimer	100	97%	1, 2
								1Z7E	B	3		100		
							4	1YRW	A	1	domains	100	96%	
								1Z7E	A	2		100		
13	P0AGD1	SODC_ECOLI	Superoxide dismutase [Cu-Zn]	173	17.68	*E. coli*	1	1ESO	A		monomer	100	87%	1, 2
							2	1ESO	A	1	domain	100	85%	
							1	2R4T	A		monomer	100	100%	
							2	2R4T	A	1	domains	100	98%	
14	P0A6U8	GLGA _ECOLI	Glycogen synthase	477	52.82	*E. coli*		2R4T	A	2		100		1, 2
							3	2R4T	A	1	domains	100	98%	
								2R4T	A	2		100		
							1	3N3R	A		monomer	66	96%	
							2	3N3R	A	1	domains	61	90%	
								3N3R	A	2		71		
							3	3N3R	AB		dimer	66	96%	

15	P13029	KATG_ECOLI	Catalase-peroxidase	726	80.02	*E. coli*	4	1U2J	A		monomer	100	41%	5
							5	1U2J	A	1	domains	100	34%	
								1U2J	A	2		100		
							6	1U2J	AB		dimer	100	41%	
							7	3WXO	A		monomer	59	96%	
							8	3WXO	A	1	domains	58	87%	
								3WXO	A	2		61		
							9	3WXO	AB		dimer	59	96%	
							10	3N3R	A	2	domains	71	98%	
								1U2J	A	2		100		
								1U2J	A	1		100		
								1C8N	A	1		21		
16	P76344	ZINT_ECOLI	Metal-binding protein ZinT	216	24.76	*E. coli*	1	5AQ6	A		monomer	100	88%	1, 2
							2	5AQ6	A	1	domain	99	62%	
							3	5AQ6	AB		dimer	100	88%	
							4	5AQ6	A	1	domains	99	94%	
								3QE1	A	1		21		
17	P0ABD3	BFR_ECOLI	Bacterioferritin	158	18.50	*E. coli*	1	2Y3Q	A		monomer	100	99%	7
							2	2Y3Q	A	1	domain	100	87%	
							3	2Y3Q	ABCD		24-mer	100	99%	
							4	2Y3Q	ABC		trimer	100	99%	
							5	2Y3Q	AB		dimer	100	99%	
18	P0AEE3	DEGS_ECOLI	Serine endoprotease DegS	355	37.58	*E. coli*	1	4RQY	A		monomer	100	84%	6
							2	4RQY	A	1	domains	100	75%	
								4RQY	A	2		100		
							3	4RQY	ABCD		24-mer	100	84%	
							4	4RQY	AB		dimer	100	84%	
							5	2R3Y	A	1	domains	100	99%	
								1TE0	A	2		100		
								2IZO	A	2		100		
19	P0AFG6	ODO2_ECOLI	Dihydrolipoyllysine-residue succinyltransferase component of 2-oxoglutarate dehydrogenase complex	405	44.01	*E. coli*	1	1E2O	A		monomer	100	57%	1, 2
							2	1E2O	A	1	domain	100	35%	
							3	1E2O	ABCD		24-mer	100	57%	
							4	1E2O	ABC		trimer	100	57%	
							5	1E2O	AB		dimer	100	57%	
							6	1E2O	A	1	domains	100	95%	
								1Y8N	B	1		32		
								1KMO	A	1		21		
								3QYF	A	2		26		
20	P0A6F5	CH60_ECOLI	60 kDa chaperonin	548	57.33	*E. coli*	1	2CGT	A		monomer	100	96%	2
							2	2CGT	A	1	domains	100	83%	
								2CGT	A	2		100		
								2CGT	A	3		100		
							3	1PCQ	A	1	domains	100	98%	
								1XCX	C	3		100		
								2CGT	H	2		100		
								4KI8	C	4		97		
								1MBM	A	3		32		
21	P0A858	TPIS_ECOLI	Triosephosphate isomerase	255	26.97	*E. coli*	1	4IOT	A		monomer	100	100%	5
							2	4IOT	A	1	domain	100	94%	
22	P0A9S3	GATD_ECOLI	Galactitol-1-phosphate 5-dehydrogenase	346	37.39	*E. coli*	1	4UEJ	B		monomer	100	100%	5
							2	4UEJ	B	1	domains	100	85%	
								4UEJ	B	2		100		
							3	4UEJ	AB		dimer	100	100%	
							4	4A2C	A	1	domains	100	95%	
								4UEJ	B	2		100		

								4NJC	F	1		19		
23	P17169	GLMS_ECOLI	Glutamine--fructose-6-phosphate aminotransferase	609	66.89	*E. coli*	1	2J6H	A		monomer	100	100%	1, 2
							2	2J6H	A	1	domains	100	95%	
								2J6H	A	2		100		
								2J6H	A	3		100		
							3	2J6H	AB		dimer	100	100%	
							4	2J6H	A	1	domains	100	100%	
								3OOJ	H	1		100		
								1MOQ	A	2		100		
								3BFM	A	2		27		
24	P0A6X3	HFQ_ECOLI	RNA-binding protein Hfq	102	11.17	*E. coli*	1	2YLB	A		monomer	100	64%	1, 2
							2	2YLB	A	1	domain	100	59%	
							3	2YLB	ABCD		hexamer	100	64%	
							4	2YLB	A	1	domains	100	91%	
								1NT0	A	2		21		
25	P0A9Q1	ARCA_ECOLI	Aerobic respiration control protein ArcA	238	27.29	*E. coli*	1	1XHE	A		monomer	100	51%	5
							2	1XHE	A	1	domains	100	92%	
								1P2F	A	2		38		
26	P0AFG3	ODO1_ECOLI	2-oxoglutarate dehydrogenase E1 component	933	105.06	*E. coli*	1	2JGD	A		monomer	100	87%	1, 2
							2	2JGD	A	1	domains	100	77%	
								2JGD	A	2		100		
								2JGD	A	3		100		
								2JGD	A	4		99		
							3	2JGD	AB		dimer	100	87%	
							4	2JGD	A	3	domains	100	98%	
								2JGD	A	2		100		
								2JGD	A	4		100		
								2JGD	A	1		99		
								2J4D	A	1		22		
								2ZHH	A	1		20		
27	P0A9A9	FUR_ECOLI	Ferric uptake regulation protein	148	16.79	*E. coli*	1	2W57	A		monomer	82	89%	1, 2
							2	2W57	A	1	domains	85	86%	
								2W57	A	2		79		
							3	2W57	AB		dimer	82	89%	
							4	2FU4	A	1	domains	100	93%	
								2W57	A	2		79		
								2WAX	B			33		
28	P03023	LACI_ECOLI	Lactose operon repressor	360	38.59	*E. coli*	1	2P9H	A		monomer	100	75%	7
							2	2P9H	A	1	domains	100	69%	
								2P9H	A	2		100		
							3	2P9H	AB		dimer	100	75%	
							4	2P9H	A	2	domains	100	98%	
								2P9H	A	1		100		
								4RZS	A	3		100		
								1UZ5	A	4		20		
29	P0ADZ4	RS15_ECOLI	30S ribosomal protein S15	89	10.27	*E. coli*	1	4A5U	B		monomer	100	90%	2
							2	4A5U	AB		dimer	100	90%	
30	P62577	CAT_ECOLX	Chloramphenicol acetyltransferase	219	25.66	*E.coli*	1	1NOC	B		monomer	100	97%	1, 2
							2	1NOC	B	1	domain	100	79%	
							3	1NOC	ABC		trimer	100	97%	
							4	1NOC	B	1	domains	100	93%	
								3GRZ	A	2		29		

31	P06720	AGAL_ECOLI	Alpha-galactosidase	451	50.66	*E.coli*	1	1S6Y	A		monomer	28	91%	5
							2	1S6Y	ABCD		tetramer	28	91%	
							3	1S6Y	ABCD		tetramer	28	91%	
							4	1S6Y	AB		dimer	28	91%	
							5	3FEF	A		monomer	25	94%	
							6	3FEF	A	1	domain	26	90%	
							7	3FEF	ABCD		tetramer	25	94%	
							8	3FEF	AB		dimer	25	94%	
							9	1U8X	X		monomer	23	96%	
							10	1U8X	ABCD		tetramer	23	96%	
							11	1U8X	AB		dimer	23	96%	
32	P0AC53	G6PD_ECOLI	Glucose-6-phosphate 1-dehydrogenase	491	55.70	*E.coli*	1	4LGV	A		monomer	38	93%	2
							2	4LGV	A	1	domain	39	90%	
								4LGV	A	2	domain	37		
							3	4LGV	ABCD		tetramer	38	93%	
							4	4LGV	ABCD		tetramer	38	93%	
							5	4LGV	ABCD		tetramer	38	93%	
							6	1QKI	A		monomer	37	97%	
							7	1QKI	A	1	domains	34	93%	
								1QKI	A	2		40		
							8	4E9I	A		monomer	37	96%	
							9	4E9I	A	1	domains	40	92%	
								4E9I	A	2		33		
							10	4E9I	AB		dimer	37	96%	
							11	1QKI	A	2	domains	40	93%	
								1QKI	A	1		34		
33	P23908	ARGE_ECOLI	Acetylornithine deacetylase	383	42.35	*E. coli*	1	3CT9	A		monomer	24	90%	2
							2	3CT9	A	1	domains	23	90%	
								3CT9	A	2		24		
							3	3CT9	AB		dimer	24	90%	
							4	4PQA	A		monomer	23	95%	
							5	4PQA	A	1	domains	12	91%	
								4PQA	A	2		26		
							6	4PQA	AB		dimer	23	95%	
							7	1VGY	A		monomer	22	95%	
							8	1VGY	A	1	domains	26	95%	
								1VGY	A	2		12		
							9	1VGY	AB		dimer	27	95%	
							10	4Q7A	C	1	domains	27	89%	
								2RB7	A	2		23		
34	P02931	OMPF_ECOLI	Outer membrane protein F	362	37.08	*E. coli*	1	2ZFG	A		monomer	100	94%	7,9
							2	2ZFG	ABC		trimer	100	94%	
							3	2ZFG	AB		dimer	100	94%	
35	P21179	CATE_ECOLI	Catalse HPII	753	84.16	*E. coli*	1	3TTV	A		monomer	100	96%	8
							2	3TTV	A	1	domains	100	66%	
								3TTV	A	2		100		
								3TTV	A	3		100		
							3	3TTV	ABCD		tetramer	100	96%	
							4	3TTV	AB		dimer	100	96%	
							5	3TTV	AB		dimer	100	96%	
							6	3TTV	A	1	domains	100	98%	
								3TTV	A	3		100		
								3TTV	A	2		100		
								1YE9	E			43		
**Proteins from yeast**														
							1	4W6Z	A		monomer	100	100%	

36	P00330	ADH1_YEAST	Alcohol dehydrogenase 1	348	36.72	*S. cerevisiae*	2	4W6Z	A	1	domains	100	86%	10
								4W6Z	A	2		100		
							3	4W6Z	AB		dimer	100	100%	
							4	2HCY	A	1	domains	100	97%	
								2HCY	A	2		100		
								5ESV	G	2		20		
37	P53184	PNC1_YEAST	Nicotinamidase	216	24.99	*S. cerevisiae*	1	3V8E	A		monomer	100	100%	13
							2	3V8E	A	1	domain	100	86%	
							3	3V8E	A	1	domains	100	100%	
								3TLX	A	2		23		
**Protein fusion tags**														
38	P0AA25	THIO_ECOLI	Thioredoxin-1	109	11.81	*E. coli*	1	3DXB	B		monomer	100	98%	1
							2	3DXB	ABCD		octomer	100	98%	
							3	3DXB	ABCD		tetramer	100	98%	
							4	3DXB	ABCD		tetramer	100	98%	
							5	3DXB	A	1	domain	100	99%	
39	P63165	SUMO1_HUMAN	Small ubiquitin-related modifier 1	101	11.56	*Homo sapiens*	1	2UYS	B		monomer	99	77%	1
40	P0ABD8	BCCP_ECOLI	Biotin carboxyl carrier protein BCCP	156	16.69	*E. coli*	1	1BDO	A		monomer	100	51%	12
							2	1BDO	A	1	domain	100	49%	
							3	1BDO	A	1	domains	100	89%	
								1IN0	A	1		27		
41	P0AFG0	NUSG_ECOLI	Transcription termination/anti termination protein NusG	181	20.53	*E. coli*	1	2XHC	A		monomer	36	96%	12
							2	2XHC	A	1	domains	51	90%	
								2XHC	A	2		19		
								2XHC	A	3		54		
								2XHC	A	4		21		
							3	4ZN3	A		monomer	23	78%	
							4	4ZN3	A	1	domains	24	74%	
								4ZN3	A	2		24		
							5	3BEZ	A		monomer	22	99%	
							6	3BEZ	A	1	domains	14	84%	
								3BEZ	A	2		16		
							7	3BEZ	ABCD		tetramer	41	99%	
							8	2XHC	A	1	domains	41	96%	
								2XHC	A	3		54		
								2V51	E			33		
42	P08515	GST26_SCHJA	Glutathione S-transferase class-mu 26 kDa isozyme	218	25.50	*Schistosoma japonicum*	1	1B8X	A		monomer	100	100%	1
							2	1B8X	A	1	domains	100	95%	
								1B8X	A	2		100		
							3	1B8X	AB		dimer	100	100%	
							4	1DUG	A	1	domains	100	95%	
								1B8X	A	2		100		
43	P0AEY0	MALE_ECO57	Maltose-binding periplasmic protein	369	40.58	*E. coli*	1	4RG5	A		monomer	100	100%	1
							2	4RG5	A	1	domains	100	96%	
								4RG5	A	2		100		
							3	3HPI	B	1	domains	100	97%	
								1HSJ	A	1		100		
							1	1HH2	P		monomer	35	69%	
							2	1HH2	P	1	domains	47	67%	
								1HH2	P	2		37		
								1HH2	P	3		22		
								1HH2	P	4		34		
							3	4MTN	A		monomer	35	81%	
								4MTN	A	1		26		
								4MTN	A	2		30		

44	P0AFF6	NUSA_ECOLI	Transcription termination/anti termination protein NusA	495	54.87	*E. coli*	4	4MTN	A	3	domains	52	74%	1
								4MTN	A	4		39		
								4MTN	A	5		24		
							5	2ASB	A		monomer	39	44%	
							6	2ASB	A	1	domains	47	43%	
								2ASB	A	2		42		
								2ASB	A	3		29		
							7	1HH2	P	1	domains	34	76%	
								1P3D	A	2		21		
								4MTN	A	2		30		
								2I1Q	A	1		33		
45	P0A3G3	DHAA_RHOSO	Haloalkane dehalogenase – HALO tag	293	33.25	*Rhodococcus sp.*	1	3G9X	A		monomer	100	99%	1
**Proteins used during purification or crystallisation**														
46	P03303	POLG_HRV14	Genome polyprotein 3C Protease	182	20.00	*Rhinovirus B*	1	3Q3Y	A		monomer	53	98%	1
							2	3Q3Y	A	1	domains	55	88%	
								3Q3Y	A	2		51		
							3	3Q3Y	AB		dimer	53	98%	
							4	3R0F	A		monomer	50	99%	
							5	3R0F	A	1	domains	50	80%	
								3R0F	A	2		49		
							6	3SJI	A		monomer	50	98%	
							7	3SJI	A	1	domains	49	79%	
								3SJI	A	2		50		
							8	3Q3Y	A	2	domains	50	87%	
								1CQQ	A	1		55		
47	P00698	LYSC_CHICK	Lysozyme C	147	16.24	*Gallus gallus*	1	2VB1	A		monomer	100	88%	1
							2	2VB1	A	1	domains	100	88%	
								2VB1	A	2		100		
							3	2VB1	A	1	domains	100	88%	
								2EKS	C	2		100		
48	P04517	POLG_TEV	TEV- Genome polyprotein	242	27.50	*Tobacco etch virus*	1	1LVM	A		monomer	99	91%	1
							2	1LVM	A	1	domains	99	71%	
								1LVM	A	2		100		
							3	1LVM	A	2	domains	99	95%	
								1LVM	A	1		100		
								3G7S	A	3		19		
49	P00735	THRB_BOVIN	Prothrombin	295	33.81	*Bos taurus*	1	5EDM	A		monomer	100	100%	1
							2	5EDM	A	1	domain	100	77%	
							3	5EDM	AB		dimer	100	100%	
							4	3NXP	A	1	domains	100	89%	
								2PGB	A			100		
50	P00742	FA10_HUMAN	Coagulation factor X	488	54.73	*Homo sapiens*	1	2Y5F	A		monomer	100	48%	1
							2	2Y5F	A	1	domains	100	42%	
								2Y5F	A	2		99		
							3	1KIG	H	1	domains	88	93%	
								1FXY	A	2		99		
								1DMR	A	1		22		
								2J95	B	1		71		
								3R8T	H	2		30		
51	Q02724	ULP1_YEAST	SUMO protease C-terminal domain	621	72.38	*S. cerevisiae*	1	1EUV	A		monomer	99	36%	1
							2	1EUV	A	1	domains	100	33%	
								1EUV	A	2		100		
							3	1EUV	A	1	domains	100	91%	
								1EUV	A	2		100		
								2HFZ	A	1		22		

								3AJA	A	1		17		
52	P98072	ENTK_BOVIN	Enterokinase	1035	114.89	*Bos taurus*	1	1Z8G	A		monomer	33	33%	1
							2	1Z8G	A	1	domains	16	32%	
								1Z8G	A	2		43		
							3	3T2N	B		monomer	30	31%	
							4	3T2N	B	1	domains	40	29%	
								3T2N	B	2		17		
							5	3K65	B		monomer	32	25%	
							6	3K65	B	1	domain	35	21%	
							7	3W94	A	1	domains	56	91%	
								2WNO	A	1		36		
								3ZSC	A	1		19		
								1H41	A	2		15		
53	P00760	TRY1_BOVIN	Trypsin	246	25.79	*Bos taurus*	1	3MFJ	A		monomer	100	91%	1
							2	3MFJ	A	1	domains	100	77%	
								3MFJ	A	2		100		
							3	3MFJ	A	1	domains	100	97%	
								3MFJ	A	2		100		
								2DLA	A	2		22		
54	P00766	CTRA_BOVIN	Chymotrypsin	245	25.67	*Bos taurus*	1	1GCT	A		monomer	100	96%	1
							2	1GCT	A	1	domains	100	80%	
								1GCT	A	2		100		
							3	1GCT	A	2	domains	100	93%	
								1GCT	A	1		100		
								3TW6	C	7		28		
55	P00800	THER_BACTH	Thermolysin	548	60.10	*Bacillus thermoproteolyticus*	1	3DNZ	A		monomer	100	58%	1
							2	3DNZ	A	1	domains	100	57%	
								3DNZ	A	2		100		
							3	3DNZ	A	2	domains	100	95%	
								3DNZ	A	1		100		
								2OUA	A	1		27		
								3DMY	A	3		26		
								4UB6	R			32		
56	P06873	PRTK_ENGAL	Proteinase K	384	40.30	*Engyodontium album*	1	2PWA	A		monomer	100	73%	1
							2	2PWA	A		domains	100	97%	
								1SCJ	B	1		38		
								2PWF	A	3		26		
57	P00791	PEPA_PIG	Pepsin	385	41.26	*Sus scrofa*	1	3PSG	A		monomer	100	95%	1
							2	3PSG	A	1	domains	99	90%	
								3PSG	A	2		99		
							3	3PSG	AB		dimer	100	95%	
							4	3PSG	A	1	domains	99	99%	
								1YX9	A	2		100		
								3G15	A	1		28		
58	P08246	ELNE_HUMAN	Neutrophil elastase	267	28.52	*Homo sapiens*	1	2Z7F	E		monomer	100	82%	1
							2	2Z7F	E	1	domains	100	72%	
								2Z7F	E	2		100		
							3	2Z7F	E	2	domains	100	94%	
								2Z7F	E	1		100		
								3P42	A	2		17		
59	P81054	PLMP_GRIFR	LysN Peptidyl-Lys Metalloendopeptidase	348	36.88	*Grifola frondosa*	1	1G12	A		monomer	100	48%	1
							2	1G12	A	1	domains	100	95%	
								2X3A	A	2		24		
								3PR5	B	4		29		

60	Q7M135	LYSC_LYSEN	Lysyl endopeptidase	269	27.96	*Lysobacter enzymogenes*	1	4NSV	A		monomer	100	98%	1
							2	4NSV	A	1	domains	100	70%	
								4NSV	A	2		99		
							3	4NSV	A	1	domains	100	93%	
								4NSV	A	2		99		
								2P6A	C	1		27		
61	P00639	DNAS1_BOVIN	DNase protein	282	31.35	*Bos taurus*	1	3W3D	B		monomer	100	92%	1
							2	3W3D	B	1	domain	100	84%	
							3	3W3D	B	1	domains	100	93%	
								3CA2	A	4		18		
**Proteins from other sources**														
62	B4SL31	B4SL31_STRM5	Alkaline phosphatase	397	40.61	*Stenotrophomonas maltophilia*	1	5JK4	A		monomer	100	94%	11
							2	5JK4	A	1	domains	100	80%	
								5JK4	A	2		100		
							3	5JK4	A	1	domains	100	89%	
								5JK4	A	2		100		
								2IPX	A	1		13		
